# SPRAYDYE: a phase I/II randomised pilot study to assess the safety and feasibility of topically applied AKRO-6qcICG, a cathepsin-activatable fluorescent imaging agent, for real-time intraoperative resection margin assessment in breast-conserving surgery

**DOI:** 10.3389/fsurg.2026.1845151

**Published:** 2026-07-22

**Authors:** Cedric M. W. Pesch, Daan G. J. Linders, Taryn L. March, Martin Pool, A. Rob P. M. Valentijn, Denise E. Hilling, Ethan Walker, Brian Straight, Sven Mieog, Gerrit-Jan Liefers, Danielle Cohen, Hans Marten Hazelbag, Marieke E. Straver, James P. Basilion, Robert Rissmann, Jacobus Burggraaf, Alexander L. Vahrmeijer

**Affiliations:** 1 Department of Surgery, Leiden University Medical Center, Leiden, Netherlands; 2 Department of Dermatology, Centre for Human Drug Research, Leiden, Netherlands; 3 Department of Clinical Pharmacy and Toxicology, Leiden University Medical Center, Leiden, Netherlands; 4 Department of Surgical Oncology and Gastrointestinal Surgery, Erasmus MC Cancer Institute, University Medical Center Rotterdam, Rotterdam, Netherlands; 5 Department of Biomedical Engineering, Case Western Reserve University, Cleveland, OH, United States; 6 Akrotome Imaging Inc., Charlotte, NC, United States; 7 Department of Pathology, Leiden University Medical Center, Leiden, Netherlands; 8 Department of Pathology, Haaglanden Medical Center, The Hague, Netherlands; 9 Department of Surgery, Haaglanden Medical Center, The Hague, Netherlands; 10 Department of Radiology, Case School of Medicine, Case Western Reserve University, Cleveland, OH, United States; 11 Leiden Academic Center for Drug Research, Leiden University, Leiden, Netherlands

**Keywords:** breast imaging <radiology & imaging, breast surgery <surgery, clinical pharmacology, clinical protocols, surgical pathology <pathology

## Abstract

**Introduction:**

The incidence of tumour-positive surgical resection margins (TPRMs) after breast-conserving surgery (BCS) remains high, ranging from 10 to 40%. A TPRM, defined as breast cancer cells at the edge of the resected specimen at pathological evaluation, implies residual tumour and necessitates re-resection or boost radiation. To prevent these additional treatments, intraoperative near-infrared (NIR) fluorescence imaging with the topically applied, fluorescently quenched, cathepsin-activatable imaging agent AKRO-6qcICG might be used to detect residual cancer in the surgical cavity and guide additional resection during BCS. Cathepsins are proteolytic enzymes that are upregulated by breast cancer (associated) cells and therefore are suitable targets for tumour imaging. *Ex vivo* validation studies have shown that topically applied AKRO-6qcICG allows for clear breast cancer visualization and the detection of TPRMs. The proposed phase I/II study in healthy volunteers and breast cancer patients will assess the local and systemic safety of a single, topical dose of AKRO-6qcICG and its feasibility for intraoperative margin assessment during BCS.

**Methods and analysis:**

A total of six healthy volunteers (Part A) and 16 breast cancer patients (Part B) will be enrolled. In Part A, AKRO-6qcICG will be topically applied randomly on drawn blisters in two doses as will the vehicle compound, and one blister will be untreated functioning as a negative control. Physician and subject will remain blinded. In Part B, a single dose of AKRO-6qcICG will be topically applied in the surgical cavity. The primary objective is, with the occurrence of treatment-emergent (serious) adverse events as primary outcome measure. Secondary outcome measures include local and systemic tolerability parameters such as wound healing, numeric rating scales of pain and pruritus, vital signs, electrocardiogram parameters, clinical laboratory tests and pharmacokinetic parameters. Among the exploratory outcome measures are the diagnostic accuracy of the imaging agent to detect residual tumour in the surgical cavity and the tumour-to-background ratio of the fluorescent signal.

**Ethics and dissemination:**

This protocol has been approved by the Medical Ethical Committee Leiden-Den Haag- Delft (METC- LDD). The protocol is registered at EU Clinical Trials Register number 2025-523166-24-00. The results of this study will be reported through peer- reviewed publications and conference presentations.

**Clinical Trial Registration:**

https://ctis.eu/trial/2025-523166-24-00?from=search

## Article summary

### Strengths and limitations of this study

- Safety is thoroughly assessed in both healthy volunteers and breast cancer patients.
- Incomplete surgical resection is a major complication with huge impact on patient's life.
- A clinically relevant endpoint will be used as the primary endpoint.
- Absence of positive tumour margins will impact the exploratory outcome of this trial.
- Systemic exposure will not be comparable between healthy volunteers and patients.

## Introduction

Breast cancer is the most commonly diagnosed malignancy and the leading cause of cancer-related death among women worldwide (1). For most patients with newly diagnosed breast cancer, curative treatment consists of breast-conserving surgery (BCS) followed by adjuvant radiotherapy (2, 3). Although BCS provides survival outcomes comparable to those of total mastectomy, it is associated with a 10%–40% risk of positive resection margins, which increase the likelihood of local recurrence and frequently necessitate re-excision surgery or boost radiotherapy (4–10). Oncoplastic breast-conserving surgery has been shown to reduce re-excision rates, but approximately 10.5% of patients still require further surgery (11). These additional interventions are also associated with increased morbidity, poorer cosmetic outcomes, a higher risk of complications, increased healthcare costs and adverse psychological effects (12). Various pathological and imaging techniques have been investigated to improve intraoperative margin assessment and reduce positive resection margin rates following BCS; however, most have significant clinical and technical limitations that have hindered their widespread adoption (13). Current intraoperative margin assessment strategies include frozen section analysis, imprint cytology, specimen radiography and intraoperative ultrasound (13). Although frozen section analysis can reduce re-excision rates, its routine implementation is limited by increased operative time, specialised pathology expertise, tissue sampling error and variable diagnostic performance across institutions. Frozen section analysis has been reported to achieve pooled sensitivity and specificity of approximately 81% and 97%, respectively, although substantial heterogeneity between studies has been observed (*I*^2^ = 90.8%) (14). An ideal method for margin assessment during BCS would swiftly, non-invasively, and in real-time detect residual malignancy on the surgical cavity surface, ideally being cost-effective and user-friendly. Intraoperative tumour-targeted near-infrared (NIR) fluorescence imaging is a promising method that could meet these criteria. This technique can highlight tumour tissue by administrating a fluorescent contrast agent that selectively binds to or is activated by tumour cells. A special NIR fluorescence (700–900 nm) camera system is used to detect the contrast agent (15).

Currently, most tumour-targeted contrast agents for fluorescence-guided BCS are administered intravenously prior to surgery (16, 17). Alternatively, contrast agents could be topically applied to the surgical cavity after tumour resection, offering fast visualization of residual tumour, smoother integration into the surgical workflow, lower chances of systemic side effects, and improved cost-effectiveness. Several topical fluorescent probes have been tested *in vivo* in a preclinical setting and *ex vivo* on resected human breast cancer specimens, but these agents were not able to adequately distinguish malignant from benign breast tissue (18, 19). A promising topically applied contrast agent for fluorescence-guided BCS is AKRO-6qcICG, a fluorescently quenched, protease-activatable probe composed of indocyanine-green (ICG) and a NIR quencher conjugated to a peptide Cbz-Phe-Lys core (20). It functions as a “smart” probe, producing a fluorescent signal only when processed by tumour-associated enzymes – cathepsin B, L, and S - which are highly upregulated in breast cancer compared to healthy tissue (21, 22). AKRO-6qcICG leverages a latent lysosomotropic effect (LLE), accumulating fluorescent fragments in lysosomes, enhancing signal strength and duration (20). In an *ex vivo* validation study AKRO-6qcICG, formulated in a thermosensitive gel, effectively differentiated between human breast cancer and adjacent healthy tissue and allowed for the detection of tumour-positive resection margins with a high sensitivity and considerable specificity (23, 24). However, due to the soft, pliable nature of a resected breast cancer specimen, its geometry does no longer accurately correspond to that of the resection cavity. Consequently, correlating an *ex vivo* detected tumour-positive resection margin to the *in vivo* location of the residual tumour that needs to be excised can be very difficult. Combining the *ex vivo* margin assessment with the *in vivo* detection of residual tumour tissue on the surgical cavity walls seems to be the most promising approach. Therefore, we propose the SPRAYDYE-study: ‘*Fa**S**t intrao**P**erative **R**esection margin **A**ssessment in breast-conserving surgery using a topicall**Y** applie**D**, fluorescentl**Y**-quenched, protease-activatable imaging ag**E**nt’.*

## Methods and analysis

### Objectives

The proposed first-in-human phase I/II study aims to evaluate the local and systemic safety of administering a single topical dose of AKRO-6qcICG in both healthy volunteers and breast cancer patients. Additionally, it will examine the feasibility of using this approach for intraoperative margin assessment during breast-conserving surgery.

### Hypothesis

It is hypothesized that a single topical dose of AKRO-6qcICG is safe and can be used to detect residual breast cancer in the surgical cavity during BCS.

### Study design

**Figure 1:**
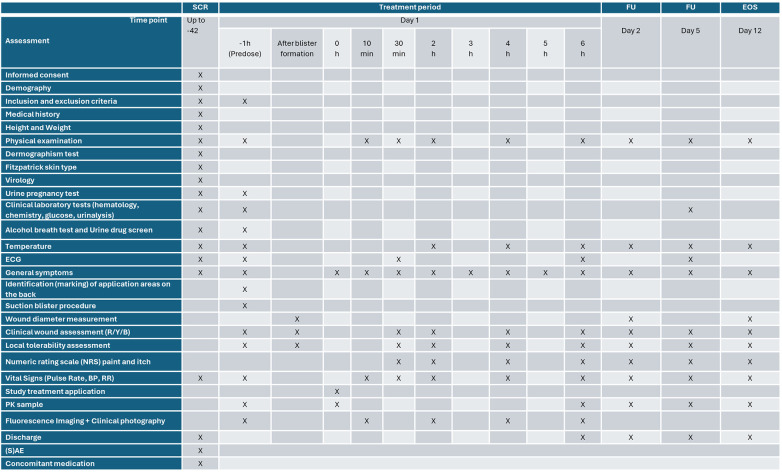
Schedule of assessments Part A..

The study consists of two parts, Part A and B. Part A of the study is a phase I, single dose, double-blind, randomized, vehicle-controlled, single-centre study in healthy volunteers (Figure 1). A total of six healthy volunteers will be included. The healthy volunteers will be evaluated for the local and systemic safety and tolerability of a single topical dose AKRO-6qcICG in thermosensitive gel vs. thermosensitive gel alone on wounded skin. In order to derisk any potential adverse events (AEs) of the compound and formulation in a patient setting, we are using the suction blister model in humans. This is routinely performed and enables systematic evaluation of local tolerability and safety signals quickly and robustly (25). Part B is a monocentre, single-dose, open-label, single arm, exploratory (proof-of-concept) phase II study in 16 breast cancer patients undergoing BCS that will evaluate the safety and feasibility of a single topical dose of AKRO-6qcICG for the detection of residual tumour in the surgical cavity.

### Setting

Part A of this study will take place at the Centre for Human Drug Research (CHDR), Leiden, The Netherlands. Part B of the study will take place in the Leiden University Medical Center (LUMC), Leiden, The Netherlands.

### Participants

For Part A, healthy volunteers, recruited through advertising, will be screened for eligibility at the CHDR screening unit. For Part B, all patients scheduled for breast-conserving surgery at the LUMC will be screened for eligibility during multidisciplinary team meetings and, when eligible for participation, informed about the study by their attending physician or specialized breast cancer nurse. Written informed consent will be obtained by investigators or dedicated staff before any study related procedure will be performed. The fundamental concepts outlined in the Declaration of Helsinki will be followed during the execution of the trial.

### Sample size

Six healthy volunteers and 16 breast cancer patients will be enrolled. As this is an exploratory study, the sample size is not based on formal statistical considerations. The proposed sample size will be sufficient to test the hypothesis.

### Patient and public involvement

Patients nor public have been involved in the design, conduct, reporting or dessimination plans of our research. The feasibility and exploratory nature of this trial and the specifics of the interventions do not facilitate an opportunity or need to do so.

### Inclusion criteria

#### Part A

Healthy volunteers must meet all of the following criteria for eligibility: they must be aged 18–55 years and demonstrate willingness and capability to adhere to study protocols. Participants must exhibit good overall health as determined by the investigator, based on comprehensive assessments including vital signs, medical history, physical examination, and laboratory evaluations. Furthermore, subjects are required to have a body mass index ranging from 18 to 32 kg/m^2^, with a minimum body weight of 50 kg. Additionally, participants must be surgically sterile, post-menopausal, or pre-menopausal with a negative urine pregnancy test at screening, and if pre-menopausal and not surgically sterile, must agree to employ effective contraception. Normal electrocardiography (ECG) and clinical laboratory findings are requisite, with any deviations considered clinically insignificant. Negative results for both drug and alcohol screenings are mandatory. Finally, a subject must have adequate healthy skin surface area on the forearm (>100 cm^2^) for study procedures.

#### Part B

Breast cancer patients must be aged 18 years or older at screening and be willing and able to adhere to study procedures. Patients must have a confirmed diagnosis of invasive breast cancer and/or grade III ductal carcinoma *in situ* (DCIS), with a total tumour diameter ≥ 5 cm, with scheduled breast-conserving surgery at the Leiden University Medical Center. Normal 12-lead ECG and clinical laboratory results are required, with any deviations deemed clinically insignificant at the investigator's discretion. Patients must be surgically sterile, post-menopausal, or pre-menopausal with a negative urine pregnancy test before receiving AKRO-6qcICG. Pre-menopausal patients who are not surgically sterile must commit to employing effective contraception for a minimum of 30 days after their final dose of study treatment. For both Part A and B, written informed consent, in accordance with ICH/GCP and national/local regulations, is obligatory prior to engagement in any study-related activities.

### Exclusion criteria

#### Part A

Exclusion criteria for Part A include any history or presence of clinically significant medical conditions or abnormalities as determined by the investigator, based on physical examination, laboratory test results (including hepatic and renal panels, complete blood count, and urine dipstick), or ECG at screening. If uncertainty arises from screening tests, additional evaluations may be conducted to confirm eligibility or deemed irrelevant by the investigator for healthy subjects. Moreover, individuals with a history of skin disease or existing skin conditions that could impede study assessments are excluded. Furthermore, subjects with tattoos, scratches, open sores, excessive hair, or skin damage in the treatment area(s) that may interfere with evaluations are ineligible. Additionally, individuals with Fitzpatrick's Skin Phototype ≥4, recent sun exposure, planned sun exposure, or use of tanning booths within four weeks prior to AKRO-6qcICG administration are excluded. Laser treatment or electrolysis on application areas within four weeks prior to administration or planned during the study period renders subjects ineligible. Shaving of the application area 72 h before administration or planned during the study period is grounds for exclusion. Likewise, recent use of topically applied treatments on the targeted area(s) within one week prior to administration is disqualifying. History of hypertrophic scarring or keloid formation, recent anticoagulant medication use, pregnancy, lactation, positive screening for hepatitis B, hepatitis C, or human immunodeficiency virus, recent prescription medication or substance use that may influence study outcomes, prior inclusion in this study, recent participation in a clinical trial, recent alcohol consumption, positive urine drug screen or alcohol test, history of anaphylactic reactions to medication or supplements, recent blood loss or donation over 500 mL, or any other condition that, in the investigator's opinion, would complicate or compromise the study or subject well-being, are grounds for exclusion.

#### Part B

In Part B, patients who exhibit a radiologic complete response of the primary tumour (rCR) following neoadjuvant therapy, those with a history of surgery and/or radiation on the ipsilateral breast, or individuals with a history of clinically significant allergies or anaphylactic reactions will be excluded. Additionally, patients with conditions that, in the investigators’ judgment, may compromise patient well-being or study objectives are ineligible. Furthermore, exclusion extends to patients with hyperthyroidism defined by TSH levels < 0.15 mU/l and free T4 levels > 24.0 pmol/l, impaired renal function defined by eGFR < 50 mL/min/1.73m2, or impaired liver function defined by values exceeding 3 times the upper limit of normal (ULN) for ALT, AST, or 2 times the ULN for total bilirubin. Participation in another clinical trial where the patient receives a fluorophore perioperatively, lactation, pregnancy, or any psychological, familial, sociological, or geographical condition that may hinder compliance with the study protocol and follow-up schedule are also grounds for exclusion.

### Study drug

AKRO-6qcICG, the sterile lyophilized Drug Product and the sterile 19.5% w/v Poloxamer-407 thermosensitive gel were manufactured in the department of Clinical Pharmacy and Toxicology, Leiden University Medical Center, according to cGMP. The study drug consists of AKRO-6qcICG Drug Product, reconstituted on the day of use in DMSO and diluted in in the thermosensitive poloxamer gel, with a final concentration of 3.5% DMSO in the study drug gel. AKRO-6qcICG is a small organic molecule whose structure is composed of both a NIR dye indocyanine green (ICG) and a non-fluorescent quencher moiety (IRDye QC-1) covalently bound to a dipeptide scaffold. 6qc-ICG is a fluorescently quenched, cathepsin-activatable imaging agent. The vehicle gel part of the study drug is composed of 19.5% w/v Poloxamer 407 solution in water for injections. Poloxamer 407 is a triblock copolymer containing a central hydrophobic polyoxypropylene block flanked by a hydrophilic polyoxyethylene (PEG) block at either end. Such poloxamers have the general formula O(C2H4O)x(C3H6O)y(C2H4O)zH. At low temperatures the polymer strands are highly solubilized by extensive hydrogen bonding to water molecules and the solution is relatively free flowing. At higher temperatures the hydrophobic fragments (polyoxypropylene blocks) aggregate to a much higher degree, leading to the exclusion of water molecules and subsequent spherical micelle formation. This results in gelation of the solution, which is reversible as the temperature decreases and the polymer strands are once again solubilized by hydrogen bonding with water molecules. The gel transition temperature is dependent on the poloxamer concentration. The concentration of 19.5% w/v Poloxamer-407 was based upon measurements of representative surgical breast cavity surface temperature during regular procedures by way of a hand-held IR temperature measurement gun (21, 22). 6qc-ICG is stable in the presence of acids, bases and metal ions.

### Intervention

Part A will include one day for screening, one treatment period of approximately 8 h and two blister evaluation visits, three and 12 days post-dosing. To mimic a surgical wound, four circular application areas of approximately 2 cm in diameter (corresponding to the wound area of approximately 1 cm in diameter and approximately 0.5–1 cm in diameter of the surrounding area) will be made on the skin of the forearm of a healthy volunteer using a blister suction device (NP-4, Electric Diversities, Maryland, USA). By using negative pressure, the device induces a 10 mm diameter painless blister in approximately 60–90 min. The roof of the formed blister is pierced with a needle and the blister fluid is aspirated. Then the blister roof, i.e., epidermal sheet, will be harvested. The four areas will be denoted as ‘Area 1’, ‘Area 2’, ‘Area 3’, ‘Area 4’. Treatments will be applied directly to the blister wounds and the surrounding skin. The four treatment conditions (AKRO-6qcICG 50 µM = 0.115 mg/l, AKRO-6qcICG 25 µM = 0.0575 mg/l, vehicle (placebo), untreated) will be randomized by computer to the four treatment areas. Both the investigator and the healthy volunteer will be blinded through sealed syringes. The assigned treatment will be applied directly onto approximately 1 cm of wounded skin and approximately 0.5–1 cm in diameter of the surrounding area. A minimum of 5 cm distance will be achieved between wounded skin. Pharmacokinetic (PK) blood and urine sample collection, local and systemic tolerability assessment, clinical wound assessment, and clinical (NIR) imaging will be performed on the study day and during the two follow-up visits.

**Figure 2:**
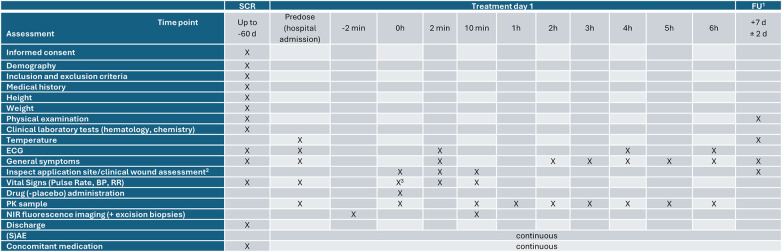
Schedule of assessments Part B.

**Figure 3:**
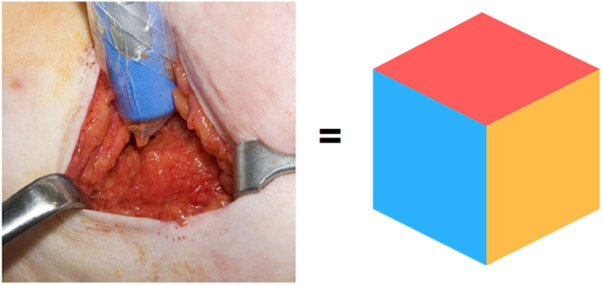
The surgical cavity simplified as a cube.

**Figure 4:**
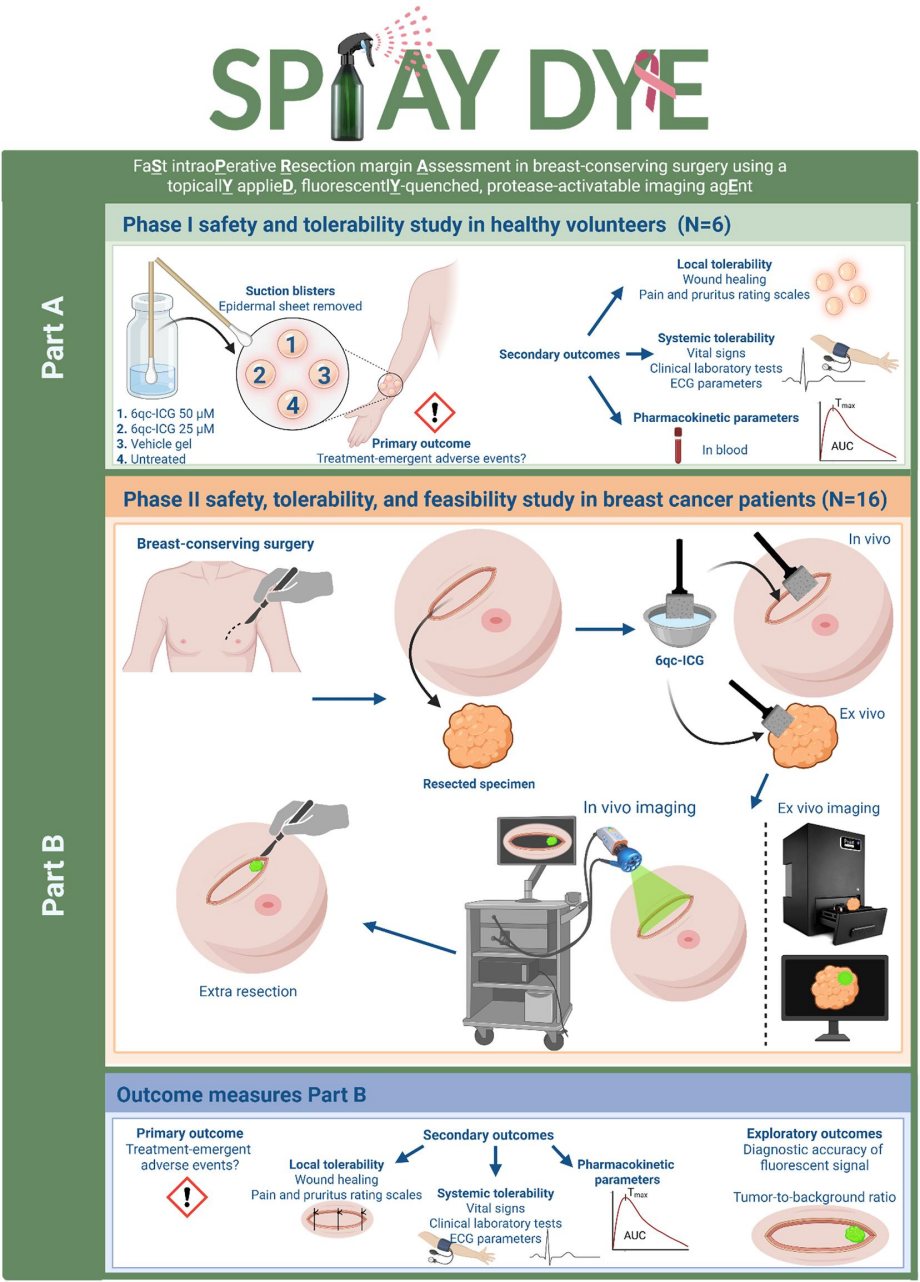
Overview of SPRAYDYE study activities.

In Part B, 10 mL of 50 μM AKRO-6qcICG in thermosensitive gel (corresponding to 1.15 mg), if well tolerated in Part A, will be topically applied onto all surgical cavity walls of all patients (superior, inferior, medial, lateral, and posterior (Figure 2)) after the standard-of-care (SOC) BCS resection. After 10 min of incubation and three washing steps with cold saline, the surgical cavity walls will be imaged with an intraoperative, clinical, open-system, NIR fluorescence-sensitive camera. The assessments will be performed in a standardised sequence and will increase the duration of the surgical procedure by a total of 15 min. At sites of fluorescence signal, biopsies will be performed to correlate the fluorescence signal to the presence of residual tumour cells. After a biopsy is taken, the surgical cavity wall will be imaged again. Additionally, in parallel AKRO-6qcICG imaging gel will be topically applied *ex vivo* onto the SOC resection specimen. After 10 min incubation and three washing steps with saline, the resected specimen will be imaged with a NIR fluorescence-sensitive camera system to detect any tumour-positive resection planes. All NIR fluorescence images will be correlated to final histopathology. On the day of surgery, blood samples will be collected for PK analysis. The complete schedule of assessments are depicted in Figures 3, 4.

### Outcome measures

#### Primary outcome

For both Part A and B, the primary outcome for the local and systemic safety and tolerability of a topical dose of AKRO-6qcICG is the occurrence of local and/or systemic treatment-emergent (serious) adverse events (TEA(S)AEs). AEs are defined as a medical occurrence in a subject to whom a medicinal product is administered. A serious adverse event (SAE) encompasses any undesirable medical incident experienced by a patient or trial participant, regardless of dosage, that leads to death, poses a threat to life, necessitates hospitalization, extends an ongoing hospital stay, causes enduring or substantial disability, or involves a significant medical event that, despite intervention, could have resulted in any of the aforementioned outcomes according to the investigator's professional judgement.

A treatment-emergent (serious) adverse event (TE(S)AE) is defined as an (serious) adverse event observed after starting administration of the specific treatment. If a patient experiences an event both prior to and after starting administration of a treatment, the event will be considered a TE(S)AE (of the treatment) only if it has worsened in severity (i.e., it is reported with a new start date) after starting administration of the specific treatment, and prior to the start of another treatment, if any.

#### Secondary outcomes

For both Part A and B, secondary outcomes include wound healing, numeric rating scales of pain and pruritus at the site of administration, changes in vital signs (pulse, blood pressure, saturation and temperature), changes in clinical laboratory tests (haematology, blood chemistry, and urinalysis), changes in ECG parameters (heart rate, PR, QRS, QT, QTcF), and PK parameters.

#### Exploratory outcomes

In Part B, exploratory outcomes include the analysis of the NIR fluorescence images of the surgical cavity walls. Diagnostic accuracy of topically applied AKRO-6qc-ICG will be determined by categorizing each surgical cavity wall (*in vivo)* and resection plane (*ex vivo*) as true positive (fluorescent and tumour positive), true negative (not fluorescent and tumour negative), false positive (fluorescent and tumour negative) or false negative (not fluorescent and tumour positive). Additionally, the signal-to-background ratio (SBR) of fluorescent areas will be calculated.

### Training

For part B, prior to their first inclusion, surgeons and other involved hospital staff of the participating centre will be trained during a site initiation visit by the principal investigator or one of the coordinating investigators. The training will include the topical application of AKRO-6qICG and the use of the clinical fluorescence camera system to image the surgical cavity walls.

### Data collection

All outcome measures will be documented in case report forms (CRFs) by the coordinating investigator or trained local research staff. The clinical data will be prospectively registered via an electronic CRF (eCRF) in a digital database of Castor EDC. Additionally, the imaging data (visible light and NIR pictures of the cavity walls and resected specimen) will be collected for postoperative analysis.

### Data validation and management

Patient data will be registered coded. The research group from LUMC will have access to all coded data in the Castor EDC database. Core data leading to published results will be accessible in a technical appendix.

### Study timeline

The estimated study start is in Q3 2026. As only a limited number of subjects will be enrolled in Part A and B, data collection is expected to be finished within one year after study start.

### Statistical analysis

Descriptive statistics will be depicted as mean (standard deviation, sd) or median (interquartile range, IQR). All statistical tests will be performed two-sided. A *p*-value of less than 0.05 will indicate a statistically significant difference. For part A and B, analysis of the safety and tolerability endpoints ((TE)(S)AEs, vital signs, ECG, and clinical laboratory tests) will be conducted with SAS 9.4 for Windows or newer (SAS Institute Inc., Cary, NC, USA). All (TE)(S)AEs will be listed and safety variable values outside the reference range will be flagged. Analysis of the pharmacokinetic endpoints (AUC_inf_, AUC_last_, CL/F, C_max_, t_1/2_, t_lag_, t_max_, Vz/F, Ae_last_, Ae_%_, CL/R) in plasma and urine will be conducted with R 3.6.1 for Windows or newer (R Foundation for Statistical Computing/R Development Core Team, Vienna, Austria, 2019). The individual PK parameters will be summarized per treatment group and, if applicable, will be presented graphically as boxplots. For Part B, the most recent version of SPSS (IBM, Armonk, New York, USA) will be used for statistical analysis of the exploratory NIR fluorescence imaging endpoints. Diagnostic accuracy analysis (including sensitivity, specificity, positive predictive value, and negative predictive value) will be performed based on a per resection wall (*in vivo* images)/per resection plane (*ex vivo* images) basis. To investigate differences in MFI between tumour tissue and adjacent healthy breast tissue on the surgical cavity walls and on the resection planes, the Wilcoxon signed-rank test will be used. The Spearman's correlation coefficient will be determined to investigate the correlation between the TBR of a fluorescent hotspot on a resection plane and the margin width at that location.

### Data monitoring

The study will be monitored for quality and regulatory compliance, by study-independent LUMC staff. Monitoring frequency will be at least annually, but may be increased depending on findings.

## Expected limitations and difficulties

Creating a representative, large surgical wound in a healthy volunteer is unethical. Instead, in Part A, four blisters will be induced on healthy volunteers, which will have a smaller total surface area compared to the surgical wound in Part B. Consequently, the total dose of AKRO-6qcICG applied to the healthy volunteers will be lower than the total dose breast cancer patients will receive in Part B. As a result, the unwanted systemic exposure measured in Part A may not fully represent that in Part B. However, using the blister model does allow for the assessment of local safety and tolerability as well as acute systemic toxicity. Additionally, it can be questioned if a detectable systemic concentration of the study drug will be achieved at all: due to the intended single-dose and topical application of the investigational product, the systemic exposure in patients in Part B is considered to be very low, especially when taking into account that the vast majority of the topically applied AKRO-6qcICG will be washed off and suctioned away after 10 min of incubation time.

The exploratory objectives of the proposed SPRAYDYE-trial - assessment of the feasibility of using AKRO-6qcICG for intraoperative margin assessment during breast-conserving surgery - may present challenges. First of all, accurate assessment of the diagnostic accuracy requires imaging sufficient surgical cavity walls containing residual tumour. To enhance the likelihood of imaging residual tumour, patients with a high risk of tumour-positive margins will be enrolled in Part B. Still, with only 16 patients enrolled, there is a possibility that none will have residual tumour, thereby preventing the calculation of sensitivity and positive predictive value. Additionally, capturing perpendicular NIR fluorescence images of all cavity walls of a breast-conserving surgery wound could pose a challenge due to the narrow space. This could be partly overcome by using a laparoscopic camera head instead of an open system.
